# Phytochemical Composition and Antimicrobial and Antibiofilm Effect of *Myrciaria cauliflora* Hydroethanolic Extract against *Staphylococcus aureus* and *Acinetobacter baumannii*

**DOI:** 10.3390/mps7040060

**Published:** 2024-08-04

**Authors:** Luciane Dias de Oliveira, Ana Luisa Monteiro Ribeiro, Sthéfani de Oliveira Dias, Geovani Moreira da Cruz, Raquel Teles de Menezes, Lara Steffany de Carvalho, Mariana Gadelho Gimenez Diamantino, Thaís Cristine Pereira, Maria Cristina Marcucci, Amjad Abu Hasna

**Affiliations:** 1 Department of Bioscience and Oral Diagnosis, Institute of Science and Technology, São Paulo State University, Campus of São José dos Campos, São Paulo 12245-000, Brazil; luciane.oliveira@unesp.br (L.D.d.O.); sthefani.dias@unesp.br (S.d.O.D.); geovani.moreira@unesp.br (G.M.d.C.); raquel.teles@unesp.br (R.T.d.M.); lara.s.carvalho@unesp.br (L.S.d.C.); thatha.this@hotmail.com (T.C.P.); cristina.marcucci@unesp.br (M.C.M.); 2 Campus São José dos Campos, Universidade Paulista—UNIP, Highway Presidente Dutra, km 157.5, South Lane, São José dos Campos, São Paulo 12240-420, Brazil; anamonribeiro@gmail.com; 3 Department of Restorative Dentistry, Endodontics Division, Institute of Science and Technology, São Paulo State University, Campus of São José dos Campos, São Paulo 12245-000, Brazil; mariana.gimenez@unesp.br; 4 School of Dentistry, Universidad Espíritu Santo, Samborondón 092301, Ecuador

**Keywords:** phytochemical composition, *Myrciaria cauliflora*, antimicrobial and antibiofilm effect, *Staphylococcus aureus*, *Acinetobacter baumannii*, herbal extracts

## Abstract

*Staphylococcus aureus* and *Acinetobacter baumannii* are opportunistic pathogens, and both are involved in different oral infections. This work aimed to analyze the phytochemical composition of *Myrciaria cauliflora* hydroethanolic extract and to evaluate its antimicrobial and antibiofilm action against *Staphylococcus aureus* (ATCC 6538) and *Acinetobacter baumannii* (ATCC 19606; multi-resistant clinical strains 58004, 50098, 566006, and H557). *Myrciaria cauliflora* hydroethanolic extract was prepared, and the content of soluble solids, flavonoids, and phenols was quantified. High-performance liquid chromatography (HPLC) was performed later. The minimum inhibitory concentration was determined using the broth microdilution method according to the Clinical and Laboratory Standards Institute, standard M7-A6, and subsequently, its minimum bactericidal concentration was determined. Then, the most effective concentrations were analyzed against biofilms. Statistical analysis was performed using the ANOVA method with Tukey’s test. The soluble solids content in the prepared hydroethanolic extract of *M. cauliflora* was 2.22%. Additionally, the total flavonoid content, measured using the quercetin standard curve, was 0.040 mg/mL. Furthermore, the total phenol content, determined using the gallic acid standard curve, was 0.729 mg/mL. HPLC analysis presented peaks of gallic acid (11.80 m), p-coumaric acid (12.09 m), cinnamic acid derivative (19.02 m), and ellagic acid (29.83 m). The extract demonstrated antimicrobial and antibiofilm action against all tested strains. However, the most effective antibacterial concentration against all the tested bacteria was 5.55 mg/mL. Therefore, these chemical components justify that *M. cauliflora* hydroethanolic extract is effective in reducing biofilm formation in *S. aureus* (standard strain) and *A. baumannii* (standard and clinical strains).

## 1. Introduction

*Staphylococcus aureus* is a Gram-positive facultative anaerobic bacterium. It is an opportunistic cocci measuring around 0.5–1.5 µm in diameter, non-endospore-forming, and generally non-encapsulated [1,2]. It resides in the oral cavity and on the skin [1,3]. *S. aureus* is implicated in various oral infections, such as endodontic, periodontal, and dental implant infections, as well as conditions like angular cheilitis [4,5,6,7,8]. More specifically, Methicillin-resistant *Staphylococcus aureus* (MRSA) is a resistant strain that poses a significant threat in both community-acquired and hospital-acquired infections as it resides in the oral cavity [1,7], leading to an increase in mortality (10–30%) [9], morbidity, and hospital stay. It has the potential to induce severe infections such as bacteremia, endocarditis, and device-related infections [10,11]. 

Device-related infections are particularly concerning as they involve the formation of biofilms on medical devices and surfaces, which are difficult to eradicate. Specific examples of such devices include indwelling catheters, prosthetic joints, cardiac pacemakers, and central venous catheters. These biofilms can form on a variety of surfaces within medical settings, such as surgical instruments, ventilators, and implantable devices, leading to persistent infections that are resistant to conventional antibiotic treatments [12,13]. It is estimated that *S. aureus* is responsible for around 20% of all hospital-acquired infections. Furthermore, its biofilm formation on medical devices poses a significant risk, particularly for patients in intensive care units, making them more vulnerable to infections [14].

*Acinetobacter baumannii* is a Gram-negative, strictly aerobic rod-shaped bacterium [15]. It is involved in endodontic and periodontal infections [16,17]. Notably, this bacterium demonstrates a remarkable ability to develop resistance to multiple antibiotics, owing to traits such as low outer membrane permeability, an efflux pump system, and the capacity to form and adhere to biofilms [18,19]. Because of its increasing resistance and status as an emerging opportunistic pathogen, *A. baumannii* is found on the list of priority pathogens for the development of new drugs [17]. Its prevalence in hospital environments is partly attributed to the formation of biofilms on hospital surfaces and devices [20]. Rapidly evolving as a nosocomial pathogen, *A. baumannii* is now recognized as a significant global health threat [21], with a mortality rate of 35% in infected hospitalized patients [22].

Biofilms are structured communities of bacteria enclosed in a self-produced extracellular matrix consisting primarily of polysaccharides. Biofilm formation represents the main mechanism of resistance and pathogenicity for both *S. aureus* and *A. baumannii* against both antibiotics and the immune responses of hosts [23]. This process involves the production of extracellular polysaccharide substances, which organize bacterial cells into clusters within multilayered structures. These biofilm formations create a protective matrix or film, shielding the microbial community from the effects of antibiotics and the host immune system [24,25]. In environments like hospitals where antibiotics are commonly used, biofilm formation is crucial for these pathogens, enhancing their ability to survive and thrive. This resilience arises from the biofilm’s capacity to impede antibiotic penetration and protect bacterial cells from immune system attacks, thereby complicating treatment and contributing to persistent infections [24,26].

The escalating antibiotic resistance displayed by these microorganisms underscores the urgent need for alternative medications and novel therapeutics to effectively combat the infections they cause. Herbal medicines, or phytotherapy, were indicated previously to combat these microorganisms and were found to be effective because of their antimicrobial action and biocompatibility [3,8,17,19]. In this context, *Myrciaria cauliflora* or *Plinia cauliflora* [27], popularly known as jaboticaba, a native Brazilian tree that belongs to the Myrtaceae family, stands out for its rich phenolic compounds with antioxidant and antimicrobial properties [28]. It has been effective against four *Candida* species in different concentrations [29] and, in a preliminary investigation, against *S. aureus* [30] because of its phenolic compounds [31] like ellagitannins and ellagic acid [32].

To the best of our knowledge, there are no studies that evaluated the antimicrobial action of *M. cauliflora* against *A. baumannii*, and the investigation into its effects on *S. aureus* remains preliminary, necessitating further research. Therefore, the aim of this study was to analyze the phytochemical composition of *M. cauliflora* hydroethanolic extract and to evaluate its antimicrobial and antibiofilm action against *S. aureus* (ATCC 6538) and *A. baumannii* (ATCC 19606) multi-resistant clinical strains (58004, 50098, 566006, and H557). The null hypothesis is that the extract has no antimicrobial action against either of the tested microorganisms.

## 2. Materials and Methods

### 2.1. M. cauliflora Hydroethanolic Extract Preparation

The extract was prepared from jaboticaba bark in which 40 g was immersed in 400 mL of hydroalcoholic solution composed of 50% ultrapure water obtained in the Milli-Q^®^ system and 50% of absolute ethanol (ethyl alcohol 99.5%—Merck, Darmstadt, Germany) for 48 h. Later, the extract was filtered and stored in a different bottle. The use of plant parts in the present study complies with international, national, and/or institutional guidelines. 

### 2.2. Content of Soluble Solids in M. cauliflora Hydroethanolic Extract

Next, the soluble solids content of the extract was quantified, in which 5 mL of the prepared *M. cauliflora* hydroethanolic extract was placed in beakers (in triplicate) and incubated in a drying oven at 60 °C until completely dry. The dried extract was then weighed to determine the percentage of soluble solids using the following equation [33]:

% soluble solids (m/V) = (m − b) × 100/Va

% soluble solids (m/m) = % soluble solids (m/V)/density

where

b = beaker mass;
m = final mass of the extract after drying;
Extract density = m/V (mass of the 5 mL aliquot weighed, and V is the volume of 5 mL).

### 2.3. Determination of Total Flavonoid Content in M. cauliflora Hydroethanolic Extract

A stock solution was prepared by placing 100 µL of the extract in a 10 mL volumetric flask and, in sequence, by adding 9900 µL of methanol in a proportion of (1:99). Subsequently, the procedure was replicated three times. From the stock solution, a 200 µL aliquot was withdrawn and transferred to a 10 mL flask already containing approximately 5 mL of methanol. Then, 200 µL of aluminum chloride (AlCl_3_) was added, and the volume was adjusted to approximately 10 mL with methanol, in a proportion of (2:2:98). The resulting solution was stirred and incubated in a water bath at 20 °C for 30 min. Later, the meniscus was adjusted, and the absorbance was measured at 425 nm using a spectrophotometer [19,34,35]. 

### 2.4. Determination of Phenol Content in M. cauliflora Hydroethanolic Extract

A different stock solution was prepared, this time, by adding 200 µL of the extract and 800 µL of Folin–Ciocalteu (F-C) reagent to a 10 mL volumetric flask containing approximately 5 mL of distilled water under agitation to obtain the stock solution (10 µg/mL). Subsequently, the procedure was replicated three times. An aliquot of 200 µL of the stock solution was transferred to a 10 mL volumetric flask (1:50) containing approximately 5 mL of distilled water, and 800 µL of the Folin–Ciocalteu (F-C) reagent was added. The resulting solution was stirred, and within 1 to 8 min, 1.2 mL of 20% sodium carbonate-tartrate buffer solution was added. The volume was completed with water until close to the meniscus, and the solution was kept in a water bath at 20 °C. After 2 h, the meniscus was adjusted to final volume at 20 °C, shaking for a few seconds, and a reading was taken at 760 nm on a spectrophotometer [19,34,35].

### 2.5. High-Performance Liquid Chromatography Analysis of M. cauliflora Extract

To analyze and measure the markers within the *M. cauliflora* hydroethanolic extract, we conducted high-performance liquid chromatography (HPLC) using an advanced liquid chromatograph equipped with a diode-array detector (HPLC-DAD) and an automated injector, specifically the D-7000 model from Merck-Hitachi (Merck KGaA, Darmstadt, Germany). The chromatographic setup utilized a mobile phase composed of a solution of water and formic acid (PA, Merck, Darmstadt, Germany) in a ratio of 95:5 (solvent A), along with high-grade methanol from Merck (Darmstadt, Germany) as solvent B. Flow was maintained at 1 mL/min, with a gradual increase in solvent B concentration from 0% to 70% over a 50 min period. Detection of compounds was carried out at wavelengths of 320 nm [19]. 

### 2.6. Antimicrobial Effect of M. cauliflora Hydroethanolic against Planktonic Forms of Bacteria

The following strains were tested: I) standard *S. aureus* strain from the American Type Culture Collection (ATCC—6538); II) standard strain of *A. baumanni* (ATCC 19606); and III) clinical strains of *A. baumanni* (58004, 50098, 566006, and H557). They were obtained from the Bioclin Laboratory of the Policlin Group (São José dos Campos—SP, Brazil). The clinical strains were previously identified by biochemical testing (Rugai, Enterokit C, Kit NF, Newprov, Pinhais, PR, Brazil) within the Santa Casa Hospital (São José dos Campos, SP, Brazil) and were collected from tracheal aspirates, wound infection, burn wound, and catheter of intensive care unit hospitalized patients [36].

To determine the minimum inhibitory concentration (MIC), the broth microdilution method was used according to the Clinical and Laboratory Standards Institute (CLSI) standard M7-A10 [37]. For this, bacterial inocula were prepared from cultures with 24 h of reactivation, where bacteria colonies were diluted in a sterile physiological solution (NaCl 0.9%), followed by standardization in a spectrophotometer (B582, Micronal, São Paulo, Brazil). *M. cauliflora* hydroethanolic extract was diluted, in which 100 µL of Mueller–Hinton (MH) broth (Himedia^®^, Mumbai, India) was added to the wells of 96-well microplates, with n = 10 for each test group. Then, 100 µL of the extract (22.2 mg/mL) was added to the first well of each test group, where 10 serial dilutions (22.2, 11.1, 5.55, 2,77, 1.38, 0.69, 0.34, 0.17, 0.08, and 0.04 mg/mL) were made. Finishing the test assembly, standardized inocula at 10^6^ CFU/mL were dispensed into all wells in a volume of 100 µL. This was the starting inoculum, which was subsequently diluted twofold in the wells, resulting in a final concentration of 10^5^ CFU/mL used in the MIC test. The hydroalcoholic vehicle (50% water and 50% absolute alcohol) was also tested to ensure that it did not have antibacterial activity. After incubation for 24 h at 37 °C, the MIC was determined in the last well of the microplate, which did not show turbidity, indicating microbial growth. This test was performed in triplicate for each strain studied. 

To determine the minimum bactericidal concentration (MBC) of the *M. cauliflora* hydroethanolic extract, an aliquot from all wells was plated on Brain Heat Infusion (BHI) agar. After incubation at 37 °C for 24 h in wells where no colony growth was observed, the MBC of the extract for the analyzed strain was determined.

### 2.7. Antimicrobial Effect of M. cauliflora Hydroethanolic Extract against Monotypic Biofilms

Biofilms were formed on the bottom of 96-well microplates. New bacteria inocula were prepared where colonies were diluted in a sterile physiological solution (NaCl 0.9%), followed by standardization at 490 and 625 nm for all tested strains of *S. aureus* and *A. baumannii*, respectively, in a spectrophotometer (B582, Micronal, São Paulo, Brazil) obtaining a concentration of 10^7^ CFU/mL. The microplate was divided into groups with n = 12, where 100 µL of the bacterial inocula were distributed into the wells, along with 100 µL of the BHI broth. The biofilms were incubated under constant agitation at 75 rpm for 48 h, with replacement of the culture medium after 24 h of incubation.

The biofilms were placed in contact with *M. cauliflora* hydroethanolic extract for 24 h at pre-determined effective MBC, which was 5.55 mg/mL and another concentration of 11.1 mg/mL. Chlorhexidine solution (CHX) at a concentration of 0.06% was used as a positive control, and BHI broth as a negative control. After treatment, the antibiofilm activity of the extract was evaluated by quantifying the metabolic activity of the bacteria using the MTT (3-4,5- Dimethylthiazol-2-yl) -2,5 Diphenyltetrazolium Bromide) test. To do this, the plates were washed twice with sterile physiological solution (0.9% NaCl), and then 200 µL of 0.5 mg/mL MTT solution was added, and the plate was incubated away from light for 1 h in an oven at 37 °C. Afterward, the solution was removed, and 200 µL of Dimethylsulfoxide (DMSO) was added, followed by incubation at 37 °C for 10 min and shaking in a shaker for another 10 min. Afterward, the optical densities (ODs) were read on a microplate reader at 570 nm and converted into a percentage of reduction in cell viability using the following formula: % Reduction in viability = 100 − (OD-treated group × 100/average of the control group). Three independent experiments were carried out, with 4 replications each, totaling n = 12 for the bacterial strain for each exposure time [19].

### 2.8. Statistical Analysis

Data from the antibiofilm test were analyzed with the Graphpad prism 5.0 software. Normality was assessed to define the appropriate test: the ANOVA method with Tukey’s test was used for results with a normal curve, and the Kruskall–Wallis method with Dunn’s Multiple Comparison test for tests without normal data distribution. Significance was 5% (*p* ≤ 0.05).

## 3. Results

### 3.1. Content of Soluble Solids, Flavonoids, and Phenols in M. cauliflora Hydroethanolic Extract

The content of soluble solids in the prepared extract of *M. cauliflora* hydroethanolic extract was 2.22%. Moreover, it was found that the total flavonoid content in *M. cauliflora* hydroethanolic extract was 0.040 mg/mL using the quercetin standard curve. In addition, the total phenol content in *M. cauliflora* hydroethanolic extract was at a concentration of 0.729 mg/mL using the gallic acid standard curve.

### 3.2. High-Performance Liquid Chromatography Analysis of M. cauliflora Hydroethanolic Extract

**Figure 1:**
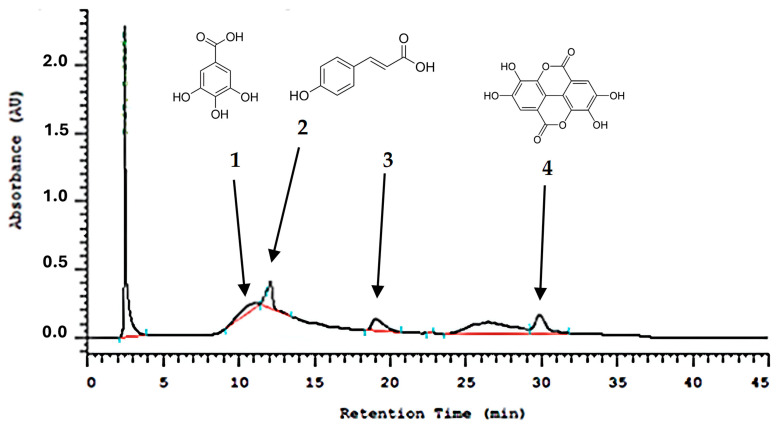
Chemical fingerprint in HPLC-DAD at 320 nm of the *M. cauliflora* hydroethanolic extract showing the peaks of the substances found.

The chromatographic analysis by HPLC presented peaks of gallic acid identified at a retention time of 11.80 min (1), p-coumaric acid identified at a retention time of 12.09 min (2), cinnamic acid derivative identified at a retention time of 19.02 min (3), and ellagic acid identified at a retention time of 29.83 min (4) (Figure 1).

### 3.3. Antimicrobial Effect of M. cauliflora Hydroethanolic Extract against Planktonic Forms of Bacteria

In this work, establishing the minimum inhibitory concentration (MIC) values proved impractical because the coloration of the extracts hindered visual assessment. However, the minimum bactericidal concentration (MBC) was 5.55 mg/mL against all tested strains of *S. aureus* and *A. baumannii*.

### 3.4. Antimicrobial Effect of M. cauliflora Hydroethanolic Extract against Monotypic Biofilms

**Figure 2:**
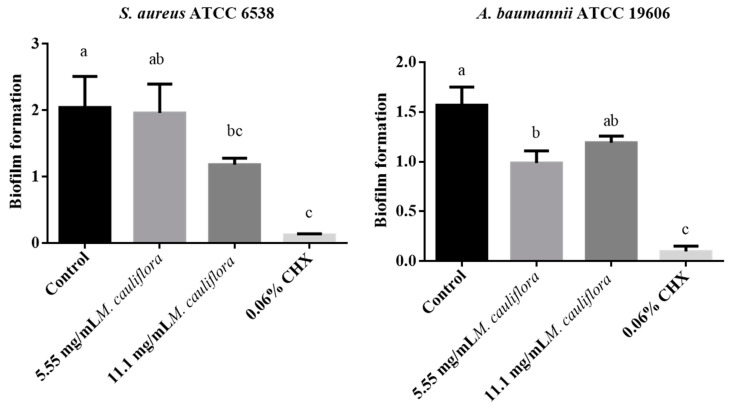
*S. aureus* and *A. baumannii* standard strains biofilm formation after treatment with 5.55 mg/mL *M. cauliflora*, 11.1 mg/mL *M. cauliflora*, 0.06% Chlorhexidine, and BHI broth (control). Different letters (a,b,c) indicate a statistically significant difference.

*M. cauliflora* hydroethanolic extract reduced the biofilm formation of *S. aureus* by 4.12% at a concentration of 5.55 mg/mL and reduced by 42.1% at a concentration of 11.1 mg/mL after 24 h (Figure 2). Both 11.1 mg/mL *M. cauliflora* hydroethanolic extract and 0.06% CHX had a statistically significant reduction in *S. aureus* biofilm formation in comparison with the control group. However, 5.55 mg/mL *M. cauliflora* hydroethanolic extract had no statistically significant difference in comparison with the control group. In addition, *M. cauliflora* hydroethanolic extract reduced the biofilm formation of the standard strain of *A. baumannii* by 44.2% at a concentration of 5.55 mg/mL and reduced by 22.5% at a concentration of 11.1 mg/mL after 24 h (Figure 2). Both 5.55 mg/mL *M. cauliflora* hydroethanolic extract and 0.06% CHX had a statistically significant reduction in *S. aureus* biofilm formation in comparison with the control group. However, 11.1 mg/mL *M. cauliflora* hydroethanolic extract had no statistically significant difference in comparison with the control group. Moreover, 0.06% CHX presented the best bacterial reduction.

**Figure 3:**
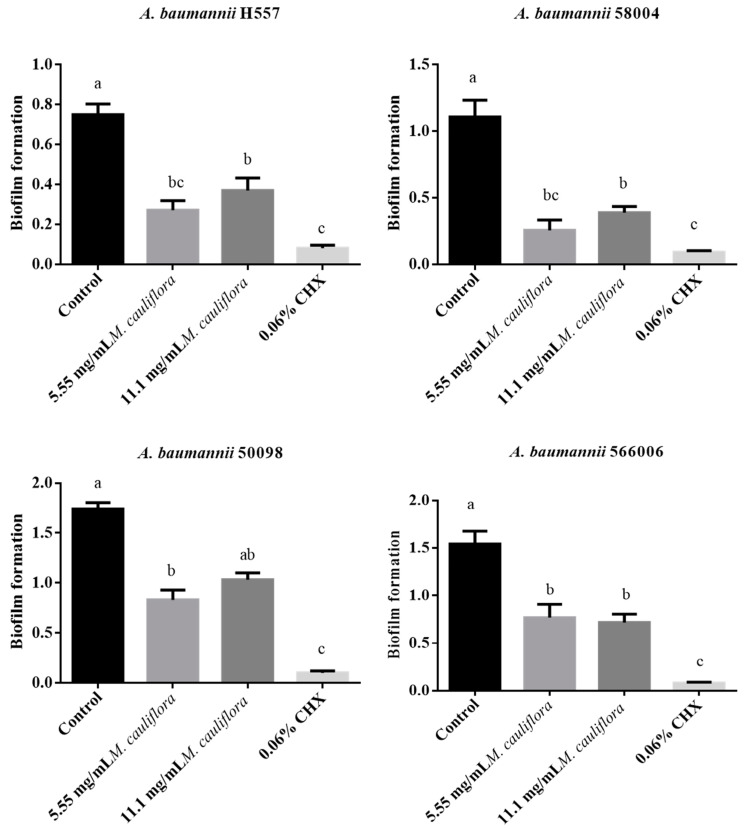
*A. baumannii* clinical strains biofilm formation after treatment with 5.55 mg/mL *M. cauliflora*, 11.1 mg/mL *M. cauliflora*, 0.06% Chlorhexidine, and BHI broth (control). Different letters (a,b,c) indicate a statistically significant difference.

Similarly, all the tested groups were effective in reducing the biofilm formation of the clinical strains of *A. baumannii* (Figure 3), highlighting that 5.55 mg/mL *M. cauliflora* hydroethanolic extract and 0.06% CHX presented the best biofilm formation reduction (70.65 to 76.40% for the extract, and 91.53% for the CHX) after 24 h against the two different clinical strains of *A. baumannii* with a statistically significant difference in comparison with the control group. *M. cauliflora* hydroethanolic extract at 11.1 mg/mL was effective in reducing *A. baumannii* biofilm formation in a percentage ranging between 51 to 64% against all tested clinical strains, except for the clinical strain 50098, where it has no statistically significant difference in comparison with the control group.

## 4. Discussion

This study was performed to more deeply investigate the antimicrobial of herbal medicines extract, as phytotherapy compounds are attracting attention because of their potential to combat multi-resistant microorganisms and are increasingly becoming an alternative treatment for various infections [38], more specifically, the hydroethanolic extract of jaboticaba against strains of *S. aureus* and *A. baumannii*, where it was found that extract is effective in reducing the biofilm formation of these microorganisms; thus, the null hypothesis of this study was rejected.

Phenolic compounds are produced by plants, and their main function is growth, development, and protection. They are secondary metabolites and play a very important role in mechanical and physiological activities. These are regulators of endogenous plant growth, with the main functions being antioxidants, structural, attractive, signaling, and protective. They act as antimicrobial, antifungal, and antioxidant agents, intercepting microbial invasions. Phenolics also play a structural role in plants and provide integrity, vigor, and vitality to plant cells. Considering that they are weak acids, phenolics such as anthocyanins, hydroxycinnamic acid, and derivatives and flavonoids have a free radical scavenging potential that is cumulative in plants under stress and prevents oxidative stress. The accumulation and biosynthesis of polyphenols in plants depend on many factors, including physiological–biochemical, molecular–genetic, and environmental factors [39]. Some factors influence the biosynthesis of phenolic compounds in plants, such as UV-B light radiation, heavy metals, amount of water, and temperature, among others [40]

The phytochemical constituents presented in plant extracts have different therapeutic actions arising from the presence of secondary plant metabolites such as flavonoids, phenols, alkaloids, tannins, glycosides, saponins, and terpenoids [41]. We analyzed this group of compounds because HPLC-DAD unambiguously detects these chemical markers. They were chosen because they present numerous biological activities, especially antimicrobial ones [42]. In the present study, the presence of total flavonoids and phenols was detected in *M. cauliflora* hydroethanolic extract, in which total flavonoid content was at a concentration of 0.040 mg/mL and the total phenol content was at a concentration of 0.729 mg/mL using the Folin–Ciocalteu method. Still, in another study, in *Plinia cauliflora*, phenols and derivatives, including gallic acid, were identified by gas chromatography coupled with mass spectrometry (GC-MS), and 27 compounds, including flavonoids, phenolic acids, tannins, and their derivatives; sugars and their derivatives; carboxylic acids and their derivatives; and alkaloids were identified using ultra-high-performance liquid chromatography–tandem mass spectrometry (UHPLC-MS/MS) [43]. The presence of flavonoids and phenols explains the antimicrobial efficacy of the extract against the tested bacteria [44]. Different methods may be used to identify the flavonoids and phenols content with different solvents, including acetone, acetone–water, ethanol, ethanol–water, methanol, methanol–water, and water [45]. In the present study, the ethanol–water solvent was used.

In the literature [46], through HPLC analysis of jaboticaba skin extracts, it was found that gallic acid showed peaks at a retention time of 6.54 min for the aqueous, ethanolic, acetone, and methanolic extracts of jaboticaba skin, and p-coumaric acid was presented with a retention time of 19.88 min in the ethanolic, acetonic, and methanolic extract of jaboticaba skin. In the present study, the same compounds were found at different retention times, with gallic acid at 11.80 min and p-coumaric acid at 12.09 min. The difference in retention times may be attributed to different vehicles or different parts of the plants used to form the extract, in which different solvents have distinct polarities and interactions with the stationary phase and analytes in HPLC, and different parts of a plant contain varying concentrations of phytochemicals and secondary metabolites. These differences can influence the interaction of compounds with the stationary phase during HPLC analysis [47,48].

Gallic acid is a phenolic component that has several pharmacological properties, such as antibacterial, anti-allergic, antioxidant, antimutagenic, anti-inflammatory, neuroprotective, and anticarcinogenic actions [49,50]. It is effective against *Escherichia coli*, *S. aureus*, and *Serratia marcescens* [49], as it can disrupt the cell membrane, altering its charge, hydrophobicity, and surface permeability [50]. This may explain the efficacy of *M. cauliflora* hydroethanolic extract against strains of *S. aureus* and *A. baumannii* in the present study.

P-coumaric acid is a phenolic acid found in the composition of several foods, such as some cereals, fruits, and legumes, and has antioxidants and anti-inflammatory actions [51]. Although natural carboxylic acids generally do not act efficiently as antibiotics [52], in another study, they demonstrated synergistic effects with antibiotics against multi-resistant Gram-negative (*E. coli*, *Enterobacter aerogenes*, and *P. aeruginosa*) and Gram-positive strains (*S. aureus*) [53] in which cinnamic, p-coumaric, and ferulic acids were the most active, combined synergistically with most antibiotics and demonstrated greater activity against all microorganisms tested. Moreover, Chen et al. [54] observed the inhibitory effect of p-coumaric acid on quorum sensing against *C. violaceum* and its potential in the preservation of pork. The combination of p-coumaric acid with potassium sorbate resulted in an inhibition of bacterial growth, offering a promising alternative to synthetic preservatives, as evidenced by the reduction in the amount of potassium sorbate used.

The antibacterial efficacy of *M. cauliflora* extract derived from 3 g of dry peels was assessed against both *S. aureus and E. coli*, presenting its ability to inhibit both Gram-positive and Gram-negative bacteria [55]. In another study against *S. mutans*, *S. sobrinus*, and *S. sanguis* in different concentrations with different vehicles [56]. In addition, in another study against *B. subitilis*, *E. coli*, *S. aureus*, *P. aeruginosa*, and *C. albicans* were found effective in different concentrations with different MIC values in inhibiting the growth of all tested microorganisms except *C. albicans* [43]. In our study, jabuticaba bark was used to produce the hydroalcoholic extract, and the MBC on the planktonic bacteria of *S. aureus* was presented at a low dose, being 5.55 mg/mL of the total value of the pure extract of 22.2 mg/mL just as in the study by Oliveira et al. [46], in which the aqueous and ethanolic extract demonstrated growth inhibition at low doses, thus probably guaranteeing less toxicity, the hydroalcoholic vehicle used in this study also presents itself as a less toxic option, being interesting for future cellular toxicity and genotoxicity tests.

To the best of our knowledge, this is the first work in the literature that evaluated the antimicrobial effect of *M. cauliflora* hydroethanolic extract against *A. baumannii* standard and clinical strains. Because of that, it was not possible to compare the present results with others in the literature. However, it is worth mentioning that *M. cauliflora* hydroethanolic extract reduced the biofilm formation of the standard strain of *A. baumannii* by 44.2% at a concentration of 5.55 mg/mL and reduced by 22.5% at a concentration of 11.1 mg/mL after 24 h. Moreover, 5.55 mg/mL *M. cauliflora* hydroethanolic extract and 0.06% CHX presented the best biofilm formation reduction (70.65 to 76.40% for the extract and 91.53% for the CHX) after 24 h against the two different clinical strains of *A. baumannii* with a statistically significant difference in comparison with the control group. *M. cauliflora* hydroethanolic extract at 11.1 mg/mL was effective in reducing *A. baumannii* biofilm formation in a percentage ranging between 51 to 64% against all tested clinical strains, except for the clinical strain 50098, where it has no statistically significant difference in comparison with the control group.

Lastly, the antimicrobial action mechanisms of *M cauliflora* can be attributed to its rich composition of bioactive compounds, particularly phenolic compounds, flavonoids, and tannins. The synergistic action of these compounds can enhance the overall antimicrobial efficacy of the plant extracts, causing cell membrane disruption, enzyme inhibition, and/or biofilm disruption of the microorganisms [55,57]. The present results encourage future studies to evaluate the *M. cauliflora* hydroethanolic extract as an endodontic irrigant/intracanal medication or mouthwash for endodontic and periodontal infections as a topic antibacterial agent or even systemically, to be used to control hospital infections caused by resistant microorganisms.

## 5. Conclusions

*M. cauliflora* hydroethanolic extract demonstrates promising antimicrobial properties as it was effective in reducing biofilm formation in *S. aureus* (standard strain) and *A. baumannii* (standard and clinical strains), highlighting its potential as a natural antimicrobial agent. The most effective antibacterial concentration against all the tested bacteria was 5.55 mg/mL. Still, further studies could focus on optimizing extraction methods and exploring the extract’s efficacy in various applications.

## Data Availability

Data are available upon request. d.d.s.amjad@gmail.com.
